# Th17 and Treg Cells in Bone Related Diseases

**DOI:** 10.1155/2013/203705

**Published:** 2013-09-25

**Authors:** Min Wang, Tian Tian, Shuang Yu, Na He, Daoxin Ma

**Affiliations:** Department of Hematology, Qilu Hospital, Shandong University, 107 West Wenhua Road, Jinan 250012, China

## Abstract

Bone-related diseases share the process of immune response that targets bone tissue and bone marrow and then induce adverse effects on structure and function. In recent years, reciprocal relationship between immune cells and bone systems has been uncovered gradually. Regulatory T (Treg) and T helper 17 (Th17) cells are newly identified subsets of CD4+ T cells, and the balance between them is particularly essential for maintaining immune homeostasis. Accumulated data have demonstrated quantitative or functional imbalance between Th17 and Treg in bone related diseases, suggesting that Th17 and Treg cells are involved in these bone diseases. Understanding the molecular mechanisms regulating Th17 and Treg cells will create opportunities for the development of therapeutic approaches. This review will present the role of Th17 and Treg cells in the inflammatory bone diseases and bone marrow malignancies and find the potential therapeutic target for immunotherapy.

## 1. Introduction

Inflammatory bone diseases share the presence of an inflammatory process that targets the bone tissue and then induces adverse effects on structure and function [1]. Chronic inflammatory bone diseases are a major health problem as they are linked to increasing disability and loss of motor function. Rheumatoid arthritis (RA) and the different forms of spondyloarthritis (SpA) are chronic inflammatory diseases with persistent activation of the immune system [2, 3]. These inflammatory bone diseases can be further defined as a group of chronic musculoskeletal disorders with common inflammatory pathways, characterized by joint organ and tissue damage, increased morbidity and mortality, and reduced quality of life. From a pathological perspective, not only changes in the immune system but also molecular and cellular pathways that determine bone tissue homeostasis and remodeling will determine the outcome of these diseases [4]. Moreover, as inflammation and alterations in immune system play an important role in hematopoietic malignancies, it is important to investigate the role of dysimmunity in bone marrow malignancies. Recently, accumulated evidence has demonstrated that T helper 17 (Th17) and regulatory T (Treg) cells imbalance plays a key role in the pathogenesis of these diseases. Th17 and Treg cells are two newly identified Th subsets, which have opposite effects on autoimmunity and inflammation. Th17 cells have a proinflammatory role and have been implicated in many inflammatory conditions in humans and mice, while Treg cells have an anti-inflammatory role and maintain tolerance to self-components by regulating the activity of effector T cells [5, 6]. Many people worldwide are affected by bone-related diseases, so understanding the molecular mechanisms of these bone diseases is crucial for developing novel drugs. This review will present the role of Th17/Treg cells in the inflammatory bone diseases and bone marrow malignancies and find the potential therapeutic target for immunotherapy.

## 2. Th17 and Treg Cells

Activated CD4+ Th cells differentiate into distinct functional subsets, characterized by heritable patterns of cytokine secretion and the expression of specific transcription factors or so-called master regulators [7, 8]. Along with classical Th1 and Th2 cells, new subsets of T cells continue to be recognized. Of these new subsets, Th17 and Treg cells have attracted tremendous attention because of their connection with inflammatory and autoimmune diseases [9].

Recently, a complete novel separate lineage of CD4+ Th cells that preferentially produce IL-17 was identified, named Th17 cells. Th17 cells differentiate from naïve T cells in the presence of TGF-*β* plus IL-6 in the mouse, or TGF-*β* plus inflammatory stimuli in the human. The inflammatory stimuli in the human setting can be IL-1*β*, IL-6, IL-21, and/or IL-23. IL-23 is dispensable for the lineage commitment of Th17 cells but is required for the growth, survival, and functions of Th17 cells [10, 11]. Soon after the discovery of Th17 cells, Ivanov et al. reported that retinoid-related orphan receptor (ROR)*γ*t is selectively expressed in Th17 cells and is required for Th17 differentiation. ROR*γ*t expression is induced by the combination of IL-6 and TGF-*β* through Stat3 [12]. Th17 cells are characterized by the production of proinflammatory cytokines, including IL-17A, IL-17F, IL-21, and IL-22. IL-17, the most important effector cytokine, is involved in promoting the expression of many proinflammatory cytokines, chemokines and mediators that contribute to inflammation. IL-21, as an autocrine regulatory factor of Th17 cells, plays a key role in inducing the differentiation of Th17 and inhibiting the function of Th1 and Treg cells [13, 14]. It has been reported that Th17 cells can recruit neutrophils and macrophages to participate in and amplify the inflammatory reaction. The overproliferation and dysfunction of Th17 cells could lead to the amplification of local inflammation, thus intensifying the tissue damage. Numerous studies have indicated that the involvement of Th17 cells in the pathogenesis of many autoimmune diseases and inflammatory conditions, such as RA, systemic lupus erythematosus (SLE), and inflammatory bowel disease (IBD) [15–17]. Therefore, Th17 cells play a critical role in host defense against certain extracellular pathogens and also contribute to various inflammatory and autoimmune diseases. In humans, therapeutics targeting Th17 cells or related cytokines may offer some new approaches to some inflammatory and autoimmune diseases [18].

CD4+CD25+Foxp3+ Treg cells are a specialized Th cell subset that is engaged in maintaining peripheral tolerance, preventing autoimmune diseases, and limiting chronic inflammatory diseases by suppressing and regulating the effector function of Th cells [19]. A number of different types of Tregs have been characterized. The most prominent types are natural Tregs (nTregs), which naturally occur in the thymus, and inducible Tregs (iTregs), which can be induced in peripheral lymphoid tissues from naïve T cells [20]. Natural Tregs are a population of CD4+ lymphocytes residing in the thymus and constitute 5–10% of the peripheral naïve CD4+ T lymphocyte pool in normal mice and humans. They play a significant role in the maintenance of immunological self-tolerance and the modulation of immune responses [21, 22]. 

Induced Tregs are found in peripheral lymphoid tissues from naïve T cells [23]. These iTregs development is driven by the release of suppressor cytokines such as IL-10 and TGF-*β* [24]. The suppressive activity of Tregs is associated with the overexpression of Foxp3, the forkhead transcription factor uniquely expressed by Treg cells, which plays a critical role in controlling the development and functions of nTreg [20]. Although the differences and similarities between these two populations are yet to be fully elucidated, both have been considered to be essential for immune homeostasis. The current notion is that nTreg cells mediate suppression in a cell contact-dependent manner, while iTreg cells predominantly mediate suppression via cytokine-dependent pathways by releasing suppressor cytokines such as TGF-*β* and IL-10 [20]. Numerous studies in animal models of autoimmunity showed that defects in CD4+CD25+Foxp3+ cells can contribute to the development of autoimmune diseases and that these diseases could be reversed by the adoptive transfer of Treg cells [25]. Therefore, Treg cells may play a crucial role in human autoimmune diseases by exerting their suppressive function, and Treg-related somatic cell therapy is considered as an intriguing new intervention for autoimmune diseases [26]. In light of the opposite functions of Th17 and Treg cells, an imbalance between them may be involved in the occurrence and development of various bone-related diseases.

## 3. Th17 and Treg Cells in Inflammatory Bone Diseases

The development and homeostasis of the vertebrate skeletal system depends on a dynamic balancing between the activities of bone-forming osteoblasts and bone-resorbing osteoclasts [27]. Tipping this balance in favor of osteoclasts formation leads to pathological bone resorption [28]. Bone loss and osteoporosis-related fractures represent one of the most important complications that may occur in patients with rheumatic diseases. Researches into the bone destruction associated with inflammatory bone diseases have highlighted the importance of the interplay between the immune and skeletal systems, and accumulated evidences has indicated that Th17 and Treg cells are both involved in the process of osteoclastogenesis [29, 30].

### 3.1. Th17 and Treg Cells in Osteoclastogenesis

Osteoclasts are large and multinucleated cells formed by the fusion of precursor cells of monocyte/macrophage lineage, and they are the only cells capable of resorbing bone [31]. The minimal essential cytokines required for osteoclast formation under basal conditions are receptor activator of NF-*κ*B ligand (RANKL) and macrophage colony stimulating factor (M-CSF). RANKL and M-CSF are produced by bone marrow stromal cells, osteoblasts and activated T cells. The co-stimulation by RANKL and M-CSF is essential for the differentiation of monocytes/macrophages into osteoclasts [32]. Aberrant regulation of osteoclast precursors (OCPs) generation, mobilization, differentiation, and activation by cytokines such as tumor necrosis factor (TNF) may have a major impact on the development and progression of inflammatory bone loss [33].

It is notable that Th1 and Th2 cells inhibit osteoclastogenesis through producing inhibitory cytokines, IFN-*γ* and IL-4, respectively. Recently, it was reported that Th17 cells and related cytokines were shown, exclusively among T-cell subsets, to have the capacity to induce osteoclastogenesis [34]. IL-17 induces RANKL on osteoclastogenesis-supporting mesenchymal cells, such as osteoblasts and synovial fibroblasts. IL-17 also enhances local inflammation by increasing the production of inflammatory cytokines including TNF-*α*, IL-6 and IL-1, which further promote RANKL expression and activity. On the other hand, Th17 cells may also contribute directly to bone loss by producing RANKL. Therefore, the infiltration of Th17 cells into the inflammatory lesion is the link between the abnormal T-cell response and bone damage [35].

Tregs have an immunosuppressive function and inhibit monocyte differentiation into osteoclast *in vitro*. In recent years, several groups have reported the inhibitory effect of Treg cells on osteoclastogenesis and bone resorption, but no consensus regarding their inhibitory mechanisms has been established. Zaiss et al. showed that CD4+CD25+Foxp3+T cells purified from mouse spleen inhibited osteoclast formation partially via IL-4 and IL-10 production but mainly through cell-to-cell contact via cytotoxic T lymphocyte antigen 4 (CTLA4) [36]. However, Kim et al. [37] and Kelchtermans et al. [38] showed that human Tregs isolated from peripheral blood mononuclear cells (PBMCs) suppressed osteoclastogenesis in a cytokine-dependent manner by the production of TGF-*β* and IL-4, not cell-to-cell dependent manner. It was reported that Tregs were expanded *in vitro* with anti-CD3 and anti-CD28 antibody-coated beads, and increased expression of osteoclastogenesis-inhibiting cytokines, especially GM-CSF, IFN-*γ*, IL-5 and IL-10 [38]. Furthermore, Luo et al. recently reported that human PBMCs-derived CD4+CD25+Treg cells suppressed osteoclastogenesis and bone resorption in a TGF-*β*1 and IL-10 cytokine-dependent manner [39]. It seems that TGF-*β*1, IL-10, and IL-4 are main cytokines that are well known to inhibit osteoclastogenesis and it is likely that Treg cells exert inhibitory effects on inflammatory associated bone destruction. Additional studies would be needed to determine how Tregs affect osteoclast-mediated bone destruction under inflammatory conditions.

### 3.2. Th17 and Treg Cells in Osteoporosis

Osteoporosis is a systemic skeletal disease, largely characterised by low bone mass and microarchitectural deterioration of bone tissue, leading to enhanced bone fragility and consequent increase in fracture risk [40]. Subsequent to achieving the optimal level of bone mineral density in early adulthood, however, the skeleton in men and women exhibits an age-associated decline of bone mass. This progression toward osteoporosis is accelerated in postmenopausal women due to declining levels of estrogen, a hormone that exhibits osteoprotective properties through promoting osteoblast synthetic activity and retarding bone resorption by osteoclasts [41]. Estrogen deficiency can lead to increased osteoclast formation, decreased levels of osteoclast apoptosis and an increased capacity of mature osteoclasts to resorb bone [39]. While estrogen can regulate skeletal remodeling by directly targeting osteoblasts and osteoclasts, it has also been reported to indirectly influence bone mass by targeting the immune system [41]. Estrogen has long been known to have an impact on CD4+ T lymphocytes physiology; however, the contribution of the Tregs and Th17 to the onset and progression of postmenopausal osteoporosis is disputed [42].

It is well known that estrogen can stimulate proliferation and differentiation of Treg cells, which have been shown to suppress osteoclast formation [43]. In a further study, Luo et al. indicated that estrogen enhances the suppressive effects of Treg cells on OC differentiation and bone resorption by stimulating IL-10 and TGF-*β*1 secretion from these cells [39]. Th17 cells are regarded as the most osteoclastogenic subset of Th cells because they produce high levels of pro-osteoclastic cytokines [44]. Tyagi et al. investigated the effect of estrogen on Th17 differentiation and demonstrated that estrogen suppressed IL-17 mediated osteoclast differentiation and estrogen deficiency induced the differentiation of IL-17 secreting Th17 cells [45]. The differentiation of Th17 is inhibited by estrogen via a direct effect mediated by estrogen receptor alpha (ERa) [46].

The data reviewed above strongly support the hypothesis that the bone loss induced by estrogen deficiency is due to a complex interplay of hormones and cytokines that converge to disrupt the process of bone remodeling [44]. Treg and Th17 cells play a pivotal role in the mechanism of estrogen deficiency-induced bone loss and may be considered as potential therapeutic targets in pathogenesis of post menopausal osteoporosis.

### 3.3. Th17 and Treg Cells in Rheumatoid Arthritis

Rheumatoid arthritis (RA) is a chronic autoimmune disease with persistent inflammation of multiple synovial joints, which results in progressive tissue destruction of bone and cartilage. The pathogenesis of this destructive disease was classically viewed as a result of Th1/Th2 imbalance characterized by enhanced Th1 response in driving RA, but recent studies in the field of immunopathology have challenged the classical paradigm for RA [47].

Accumulated researches have demonstrated that both Th17 and Treg subsets play an opposite but vital role in the pathogenesis of RA. It was reported that RA patients revealed an obvious increase in peripheral Th17 frequencies and Th17-related cytokines (IL-17, IL-23, IL-6, and TNF-*α*) levels, while there is a significant decrease in Treg frequencies and Treg-related cytokine (TGF-*β*1) level [47]. Furthermore, in another study, Th17 as well as IL-17 in RA synovial fluid (SF) was found to be significantly higher than that in RA peripheral blood (PB) and normal SF [48]. In addition, several other studies have evaluated the tissue distribution of IL-17 in RA, though discrepancy existed, and mostly agreed that IL-17 are elevated in the SF, synovial tissue (ST), and PB in RA patients, suggesting the potential role of IL-17 in the development of RA [49]. IL-17 induces RANKL expression by synovial fibroblasts and osteoblasts to indirectly drive bone erosion and to activate synovial macrophages to secrete the known osteoclastogenic factors TNF and IL-1*β* [50, 51]. Furthermore, upregulated IL-17 and TNF-*α* can exert synergistic effects on stimulating synovial fibroblasts and epithelial cells to secrete proinflammatory cytokines such as IL-6, IL-8, and prostaglandin E2 and neutrophil chemokines, which mediate tissue infiltration and inflammation [47]. All of these effects together lead to joint and cartilage destruction and bone resorption in RA. More recently, Kim et al. compared Th17 and related cytokines between active and inactive RA and demonstrated that elevated levels of Th17 cells in circulation were associated with disease activity [52]. Another study reported that the frequency of Th17 cells correlated with markers of disease activity in RA, such as C-reactive protein (CRP) and tender joint count. Moreover, IL-17 expression levels correlated with poor prognosis and greater joint destruction [49, 53].

CD4+CD25+ Treg cells have a central role in protecting an individual from autoimmunity. An experimental model of collagen-induced arthritis (CIA) is exacerbated by depletion of Treg cells [54]. Several analyses of Treg numbers in the peripheral blood of subjects with RA have produced differing results. Cao et al. reported that Treg population in PB has shown no difference from that of controls, while Tregs in synovial fluid were significantly higher [55]. On the other hand Lawson et al. showed a modest decrease in Treg cells in untreated patients with early stage RA and made a conclusion that early RA is associated with a deficit in the CD4+CD25 high Treg cells [56]. However, these two findings contrasted with observations by Han et al. who reported an increase in the relative and absolute numbers of Treg cells in the PB of RA patients [57]. Despite these differences, there is a general agreement that the percentage of Treg is higher in the synovial fluid of RA patients than that in controls. Furthermore, analysis of Foxp3 mRNA and protein expression also supported the conclusion that the CD4+CD25+ Treg population isolated from RA patients were indeed abundant [58–60]. A potential explanation for the enrichment of Treg cells in arthritic joints is that the expression of specific pattern of chemokine receptors leads to preferential trafficking of Treg cells to the disease site, so the number of Treg in PB is relatively lower [61]. Although the frequency of CD4+CD25+ Treg cells is higher in the SF than that in PB of RA patients, there is still persistent inflammation in the joint, suggesting that the Treg cells are ineffective to control inflammatory responses [62]. There is increasing evidence that the suppressive function of these Treg cells is defective. One group reported a striking defect in the capacity of Tregs from RA patients to suppress effector T cell proliferation [63]. Ehrenstein et al. identified a focal defect in their ability to suppress the production of two principle proinflammatory cytokines, IFN-*γ* and TNF, by effector T cells [64]. Subsequent studies by this group have shown that this defect is associated with decreased surface expression of CTLA4 on Tregs and this defect can be reversed by overexpression of CTLA4 in these Treg cells [65]. It also showed that activated responder T cells from SF were more resistant to suppression by Treg cells than responder T cells isolated from PB [66]. However, resistance of effector T cells to suppression has not been tested in RA and was not the reason for the impaired suppression [64]. Due to the impaired regulation of Treg cells in RA, adoptive transfer of CD4+CD25+ Foxp3+ T cells could be an effective treatment.

Taken together, the imbalance of Th17 and Treg cells exerts a significantly critical impact on the process of RA, and targeting these two T cell lineages and/or its osteoclastic factors may provide alternative strategies to treat inflammatory joint diseases.

### 3.4. Th17 and Treg Cells in Periodontitis

Periodontitis is a chronic bacterial infectious inflammatory disease that affects the gingival and the bone supporting the teeth. Bacterial plaque stimulates the host inflammatory response leading to tissue damage and bone resorption. Despite many studies in the past years focused on bacterial plaque, osteoimmunology, as a cause of periodontitis, attracts more attention nowadays [67]. The bacterial biofilm is the initial factor of periodontitis, but the major periodontal tissue destruction might result from a series of immune inflammatory reactions in response to bacteria [68]. Periodontitis is one of the most important causes of tooth loss and the most prevalent form of bone pathology. In contrast to most other diseases in the field of osteoimmunology, such as osteoporosis or RA, the immune system has a dual role in the pathology of periodontitis. Immunological response is destructive and responsible for tissue degradation, while it is also necessary to control the infection and the bacterial invasion adopting a protective attitude [69]. In the past 20 years, the pathogenesis of periodontitis was classically viewed as Th1/Th2 paradigm, which cannot accurately elucidate the pathogenesis of periodontal disease. Recently, it has been studied that Th17 and Treg cells have been found to play major roles in periodontal disease [68].

Th17 and Th17-related cytokines have been demonstrated to take part in the inflammatory process of periodontitis. It was reported that Th17 cells were found infiltrated in periodontal tissues in chronic periodontitis patients, and IL-17 level in gingival crevicular fluid was significantly increased [70]. Gingival concentrations of IL-23, IL-17, IL-6, IL-1*β*, and TNF-*α* were significantly higher at severe clinical attachment loss (CAL) sites than those at normal-slight CAL sites [71]. Recently, a study reported that after nonsurgical periodontal therapy, the levels of IL-17 and IL-21 were downregulated, and the expression of ROR*γ*t was decreased correspondingly [69]. IL-17 as a pro-inflammatory cytokine enhances osteoclastogenesis by amplifying the pro-inflammatory loop of IL-1*β*, IL-6 and TNF-*α* expression and by increasing the level of RANKL and matrix metalloproteinases (MMPs) [69]. Moreover, IL-17 seems to play an important role in the mobilization and activation of neutrophils after pathogenic attack [72].

In addition to Th17, Treg infiltration may play a role in periodontal disease. Immunohistological and gene expression study has shown increased Tregs and Foxp3 in periodontitis [73]. Kobayashi et al. showed that the CD4+CD25+Foxp3+ T cells were significantly increased in the later stage of infection with *P. gingivalis*. Of importance, intracellular IL-10 analysis showed a higher frequency of IL-10-producing CD4+ T cells in inflamed gingiva than that in normal tissue. These results suggest that there are potential roles for Treg cells during the chronic stage of periodontitis in the downregulation of inflammatory responses through their IL-10 production [74]. The mediating and protective role of Treg cells was confirmed in experimental periodontitis and their anti-inflammatory property does not influence defense mechanisms against bacterial attack [75]. Furthermore, Tregs can inhibit osteoclast formation via IL-10 and TGF-*β*1 signaling pathways [39]. Therefore, Treg cells which are present in periodontal tissue represent a protective Th subset by their anti-inflammatory and antiresorptive properties. 

Thus, the above evidence demonstrates that early inflammatory responses in periodontal disease initiate CD4+ effector T cells activation, followed by Th17 and Tregs induction that may contribute to the chronic stage of periodontitis [74]. Further studies are needed to test this hypothesis and to determine the role of the two subsets in periodontal inflammation.

## 4. Th17 and Treg Cells in Bone Marrow Malignancies

In addition to providing mobility and protection for vital organs, the skeleton also serves as an essential environment in which hematopoiesis occurs. Hematopoietic stem cells (HSCs) are maintained in the bone marrow and the stromal cells that reside within the bone marrow and line the endosteal surfaces provide a niche rich in growth factors, cytokines, and adhesion molecules that sustain the pluripotent state and self-renewal of HSCs. However, the fertile microenvironment within the bone marrow can also provide a niche for the development and proliferation of certain neoplastic diseases, including hematological malignancies and metastatic solid tumors [76]. Accumulated evidences demonstrates that active function and trafficking of immune cells are observed in the bone marrow, suggesting that bone marrow is an immune regulatory organ, and the immune cells in the bone marrow may play an important role in bone marrow-mediated immunity and may serve as a therapeutic target for bone marrow-related diseases [77].

Accumulating data demonstrated that both Th17 and Treg cells are involved in the occurrence and development of various solid tumors, and the balance between Treg and Th17 cells is particularly essential for maintaining homeostasis of antitumor immunity [78]. Contrary to solid tumors, there are few studies regarding the role of Th17 and Treg cells in hematological malignancies [79].

### 4.1. Th17 and Treg Cells in Multiple Myeloma

Multiple myeloma (MM) is a relatively common hematological malignancy characterized by the clonal proliferation of malignant plasma cells in bone marrow compartment, secretion of monoclonal immunoglobulin, and suppression of normal immunoglobulin production and hematopoiesis. Although multiple myeloma is a neoplasm of B lineage differentiated cells, a complex range of numerical, phenotypic, and functional abnormalities within the T cell compartment of patients is well recognized [80]. However, the number and function of Treg and Th17 cells in MM patients are controversial.

Tregs play an important role in decreasing the host response to tumors as they have been reported to be increased in many malignancies [81, 82]. However, there are significant disagreements in the literature concerning Treg numbers and function in myeloma. Prabhala et al. demonstrated that Tregs were significantly reduced in monoclonal gammopathy of undetermined significance (MGUS) and MM. These Treg cells were described as dysfunctional and unable to suppress the proliferation of T lymphocytes in an organized manner [83]. In contrast, other authors found that they were increased in MGUS as well as in untreated and treated MM [84]. Additionally, another study did not find significant difference of the number and function of Treg cells in the peripheral blood or bone marrow between normal individuals and MM patients [85]. The conflicting results for Treg cells in myeloma may be due to the different purification techniques utilized.

More recently, in bone marrow from patients with myeloma, Dhodapkar et al. demonstrated a higher proportion of Th17 cells [86]. In addition, another study indicated the increased Th17 cells in MM patients promote myeloma cell growth and dysregulate immune function both *in vitro* and *in vivo* via IL-17 receptors (IL17R) and inhibited Th1 responses [87]. It has also been reported that Th17 but not Treg cells mediate the bone marrow infiltrating lymphocytes of patients with myeloma [88]. Interestingly, interactions between MM cells and the bone marrow microenvironment lead to production of a number of cytokines and chemokines with immunomodulatory activity that may skew Th subsets toward Th17 cells. The interplay between TGF-*β* and IL-6 which are both expressed at high levels in MM bone marrow may affect generation of Th17 cells both directly and via other proinflammatory cytokines and thereby modulate antitumor immune responses [89, 90]. Recently, Shen et al. also demonstrated significant increase of Th17 cells in patients with MM, together with increased Th17-associated cytokines (IL-6, IL-17, IL-1*β*, and IL-23) [91]. The increased concentration of Th17-related cytokines together with the increased Th17 frequencies may contribute to the pathogenesis of MM and may be an important therapeutic target for anti-MM activity. 

### 4.2. Th17 and Treg Cells in Myelodysplastic Syndromes

Myelodysplastic syndrome (MDS) is a heterogeneous group of HSCs disorders characterized by immune mediated bone marrow failure and leukemic progression in the early (E-MDS, low/intermediate-1 risk) and late stage (L-MDS, intermediate-2/high risk) of the disease, respectively. An important mechanism in BM failure in this syndrome is the increased apoptosis of hematopoietic progenitors and their progeny [92]. Various autoimmune phenomena have been reported in MDS and indicate that autoimmune responses may play a role in the pathogenesis of MDS [93]. Although many clinical and experimental data suggest the involvement of T lymphocytes in the pathogenesis of MDS, the actual effect exerted by Th cells in this scenario is yet to be unsettled [94]. Among different subsets of CD4+ T cells, Th17 and Treg cells have been shown to have a significant correlation with MDS stage and risk of disease progression [95]. MDS ranged from cases with low-risk disease and minimal blast cell number to others who present with advanced disease and rapid disease progression to AML [96]. The pathophysiology of such disease subtypes is likely to be quite disparate. As such, these two subsets of CD4+ T cells are briefly reviewed here.

As Tregs are known to influence autoimmunity and tumor progression, both of which seem to play a crucial role in MDS pathogenesis, evaluating them in this context could potentially offer new insights into the biology of these disorders. Kordasti et al. showed a significant increase of polyclonal CD4+CD25 high Foxp3+ Tregs in PB of high-risk MDS compared with low-risk or healthy age-matched donors. By contrast, in low-risk MDS, the Treg population tended to be lower, thereby permitting the emergence of autoimmune responses, including those directed against the dysplastic clones [97]. Therefore, they suggested that Tregs expansion was a feature of high-risk MDS and progression to aggressive subtypes of the disease. Moreover, a significant correlation was demonstrated between increased number of Tregs and several indicators of disease activity [97]. Subsequently, Kotsianidis et al. found that Tregs were dysfunctional, and their bone marrow homing through the CXCL12/CXCR4 axis was seriously impaired as a result of CXCR4 downregulation in E-MDS. Conversely, in L-MDS, Tregs were systemically and locally expanded and retained their normal function and migratory capacity. Moreover, the decreased Tregs were also noted in patients responding to treatment for MDS [98].

Therefore, these studies support the hypothesis that the decreased number and impaired function of Tregs may facilitate the emergence of autoimmunity in E-MDS, whereas the increase of Tregs in L-MDS may permit the expansion of leukemic clones [96]. Bouchliou et al. demonstrated for the first time that Tregs displayed deficient proliferative capacity during the E-MDS, while in L-MDS, Treg proliferation returned to normal levels. Thus, the recovery of Tregs proliferative activity might represent a candidate mechanism for the observed Tregs expansion in L-MDS. Despite being incompletely understood, the mechanisms of Tregs expansion encompass increased migration to tumor sites, induction of adaptive Tregs from CD4+CD25− T effectors, and local activation and proliferation of Tregs [99].

Currently, there are two reports address discrepant ideas that Th17 cells operate to regress or enhance leukemic progression of MDS. Kordasti et al. showed that Th17 cells were markedly increased in low-risk MDS compared with high-risk MDS, and an inverse relationship between Th17 and Treg cells was also described. The Th17: Treg ratio was significantly higher in low-risk disease compared with high-risk MDS and was correlated with increased bone marrow apoptosis. The “unfavorable” Th17: Treg ratio may explain the higher risk of autoimmunity and the improved response to immune suppression in low-risk MDS compared with high-risk MDS. The level of apoptosis was higher in bone marrow of low-risk patients with higher Th17 and related proinflammatory cytokines expression, and there was also a linear correlation between the number of Th17 and apoptosis, suggesting that Th17 may potentially induce apoptosis then ultimately bone marrow failure [97]. Therefore, it suggested that bone marrow failure in low-risk MDS may occur as a consequence of increased apoptosis induced by the inflammatory environment mediated mainly by Th17 and related proinflammatory cytokines [98]. In contrast, Bouchliou et al. observed decreased number and hypofunctionality of bone marrow Th17 cells in E-MDS patients but increased number of functionally competent bone marrow IL-17+ cells in L-MDS patients. They observed that Th17 displayed a reduced capacity to produce IL-21 and IL-22 in E-MDS and was remarkably upregulated during disease progression. Th17 effector function at the single cell level appears to be compromised during E-MDS, whereas in L-MDS patients the cytokine secretion of Th17 cells is substantially increased, indicating a generalized compromise in the Treg/Th17 axis during the early MDS stage [99].

Collectively, a clear difference exists between low-risk and high-risk MDS in terms of their immunological environment and the phenotypes of their CD4+ T cells, suggesting that high, and low-risk MDS should be considered as distinct disorders. These findings open a new arena within MDS immunobiology research which will ultimately help clinicians identify those patients who are more likely to respond to immune-modulating agents.

### 4.3. Th17 and Treg Cells in Acute Myeloid Leukemia

Acute myeloid leukemia (AML) is the most common hematological malignancy in adults, characterized by distorted proliferation and development of myeloid cells and their precursors [100]. Although the involvement of immune system impairment in the pathogenesis of AML has long been recognized, the explicit immune mechanism, especially Th17 and Treg cells, remains unknown [101].

Though recent studies suggested that Th17 cells may play a role in the pathogenesis of many human solid tumors [101, 102], there are few studies about Th17 cells in AML. Wu et al. suggested a role of Th17 cells in the pathogenesis of AML. They observed that Th17 levels along with IL-17 levels were significantly increased in peripheral blood of untreated AML patients, and reduced when patients achieved complete remission after chemotherapy, suggesting that measurement of Th17 may have clinical value in the evaluation of therapeutic effect [103]. However, another study reported that circulating Th17 cells showed no difference between healthy individuals and AML patients [104]. Therefore, the precise involvement of Th17 cells in AML pathogenesis remains unsettled and needs to be clarified in the future.

In comparison with solid malignancies, relatively little information is available about functional characteristics of Treg or their clinical significance in AML patients. It was reported that suppressive Tregs contribute to a defective antileukemic immune response in the development and persistence of AML [105]. Wang et al. have shown an increased Treg in the peripheral blood of AML patients and a concomitant elevation in bone marrow [106]. Szczepanski et al. demonstrated that circulating Tregs were significantly higher and their phenotype was distinct in AML patients relative to normal controls. They also showed that Treg-mediated suppression was mainly by IL-10 and TGF-*β*1 production as well as cell-to-cell contact. Patients with lower Treg frequency at diagnosis had a better response to induction chemotherapy. Interestingly, during the post-induction period, Treg frequency and suppressive activity remained elevated in complete remission, suggesting that Tregs were resistant to conventional chemotherapy [107]. Therefore, increased Tregs are found in AML patients and are associated with poor prognosis, and the depletion of Tregs improves outcomes [105]. On the basis of these studies, manipulation of Tregs may become a part of AML treatment in the future.

## 5. Conclusion

Th17 and Treg cells are two mutually contradictory T cell subsets. Th17 cells are identified as the major cell type involved in orchestrating tissue inflammation and autoimmunity, while Tregs play a central role in maintaining immune tolerance by suppressing immunoreactions. It is important to maintain an appropriate balance between Th17 and Treg cells that can ensure effective immunity while avoiding inflammatory and autoimmune diseases. Accumulated evidence has demonstrated quantitative or functional imbalance between Th17 and Treg in bone-related diseases, suggesting that Th17 and Treg cells are involved in these bone diseases. A better understanding of the molecular mechanisms regulating Th17 and Treg cells will create opportunities for the development of therapeutic approaches for bone-related diseases.
